# Glutamate-induced and NMDA receptor-mediated neurodegeneration entails P2Y1 receptor activation

**DOI:** 10.1038/s41419-018-0351-1

**Published:** 2018-02-20

**Authors:** Ana P. Simões, Carla G. Silva, Joana M. Marques, Daniela Pochmann, Lisiane O. Porciúncula, Sofia Ferreira, Jean P. Oses, Rui O. Beleza, Joana I. Real, Attila Köfalvi, Ben A. Bahr, Juan Lerma, Rodrigo A. Cunha, Ricardo J. Rodrigues

**Affiliations:** 1 CNC—Center for Neuroscience and Cell Biology, University of Coimbra, 3004-504 Coimbra, Portugal; 2 Institute for Interdisciplinary Research, University of Coimbra, 3030-789 Coimbra, Portugal; 3 Biotechnology Research and Training Center, University of North Carolina-Pembroke, Pembroke, NC 28372 USA; 4 Instituto de Neurociencias, Centro mixto de la Universidad Miguel Hernández de Elche y el Consejo Superior de Investigaciones Científicas, 03550 San Juan de Alicante, Spain; 5 Faculty of Medicine, University of Coimbra, 3004-504 Coimbra, Portugal

## Abstract

Despite the characteristic etiologies and phenotypes, different brain disorders rely on common pathogenic events. Glutamate-induced neurotoxicity is a pathogenic event shared by different brain disorders. Another event occurring in different brain pathological conditions is the increase of the extracellular ATP levels, which is now recognized as a danger and harmful signal in the brain, as heralded by the ability of P2 receptors (P2Rs) to affect a wide range of brain disorders. Yet, how ATP and P2R contribute to neurodegeneration remains poorly defined. For that purpose, we now examined the contribution of extracellular ATP and P2Rs to glutamate-induced neurodegeneration. We found both in vitro and in vivo that ATP/ADP through the activation of P2Y1R contributes to glutamate-induced neuronal death in the rat hippocampus. We found in cultured rat hippocampal neurons that the exposure to glutamate (100 µM) for 30 min triggers a sustained increase of extracellular ATP levels, which contributes to NMDA receptor (NMDAR)-mediated hippocampal neuronal death through the activation of P2Y1R. We also determined that P2Y1R is involved in excitotoxicity in vivo as the blockade of P2Y1R significantly attenuated rat hippocampal neuronal death upon the systemic administration of kainic acid or upon the intrahippocampal injection of quinolinic acid. This contribution of P2Y1R fades with increasing intensity of excitotoxic conditions, which indicates that P2Y1R is not contributing directly to neurodegeneration, rather behaving as a catalyst decreasing the threshold from which glutamate becomes neurotoxic. Moreover, we unraveled that such excitotoxicity process began with an early synaptotoxicity that was also prevented/attenuated by the antagonism of P2Y1R, both in vitro and in vivo. This should rely on the observed glutamate-induced calpain-mediated axonal cytoskeleton damage, most likely favored by a P2Y1R-driven increase of NMDAR-mediated Ca^2+^ entry selectively in axons. This may constitute a degenerative mechanism shared by different brain diseases, particularly relevant at initial pathogenic stages.

## Introduction

The different brain disorders present characteristic etiologies and phenotypes, and are underlined by distinctive pathogenic mechanisms. Yet, both acute and chronic brain diseases present glutamate excitotoxicity as a key pathogenic event in the development of neurodegeneration^1–3^. Glutamate-induced neuronal damage involves an abnormal Ca^2+^ influx mainly mediated by NMDA receptors (NMDARs)^4,5^. This Ca^2+^ overload then leads to the activation of calpains and other proteases mediating cytoskeleton damage^6^, paralleled by reactive oxygen species generation, mitochondrial dysfunction, and subsequent neuronal apoptosis^7–10^.

Another event systematically occurring in different brain disorders is the increase of the extracellular ATP levels, which is now recognized as a danger and harmful signal in the brain^11^, as heralded by the ability of ATP P2 receptors (P2Rs) to affect different brain pathologies^12^. In particular, there is growing evidence that the antagonism of P2X7R affords neuroprotection in a wide range of brain disorders mainly through the control of neuroinflammation^13^. Attention has also been directed to P2Y1Rs as their antagonism affords neuroprotection namely in ischemia^14,15^ or trauma^16^. P2Y1R-mediated control of brain damage has been attributed to the control of astrocytes^16,17^. However, P2Y1Rs are also located in neurons and targeted to synapses where they can modulate neuronal function through the control of neurotransmitter release^18,19^, NMDARs^20^, or calcium and potassium channels^21,22^. This P2Y1R-driven neuromodulation seems to be prominent in pathological conditions^23,24^, and was shown to control neuronal damage in ischemia^15^. The contribution of ATP to neuropathological conditions may further entail other P2Rs^12^. Thus, there is now compelling evidence for the contribution of extracellular ATP and P2Rs to the pathogenesis of a wide range of neurological disorders. Yet, the mechanisms through which extracellular ATP and P2Rs contribute to neurodegeneration remains ill defined. We now focused in a process common to different brain disorders, glutamate-induced neurotoxicity, to examine the contribution of extracellular ATP and P2Rs to neurodegeneration. We found in vitro and in vivo that extracellular ATP/ADP through the activation of P2Y1R catalyzes glutamate-induced synaptic loss and latter neuronal death in the hippocampus and the revealed underlying mechanism of action may constitute a new common mechanism in brain diseases.

## Results

### Extracellular ATP and P2Y1R activation are required for glutamate-induced degeneration of rat hippocampal neurons

**Fig. 1:**
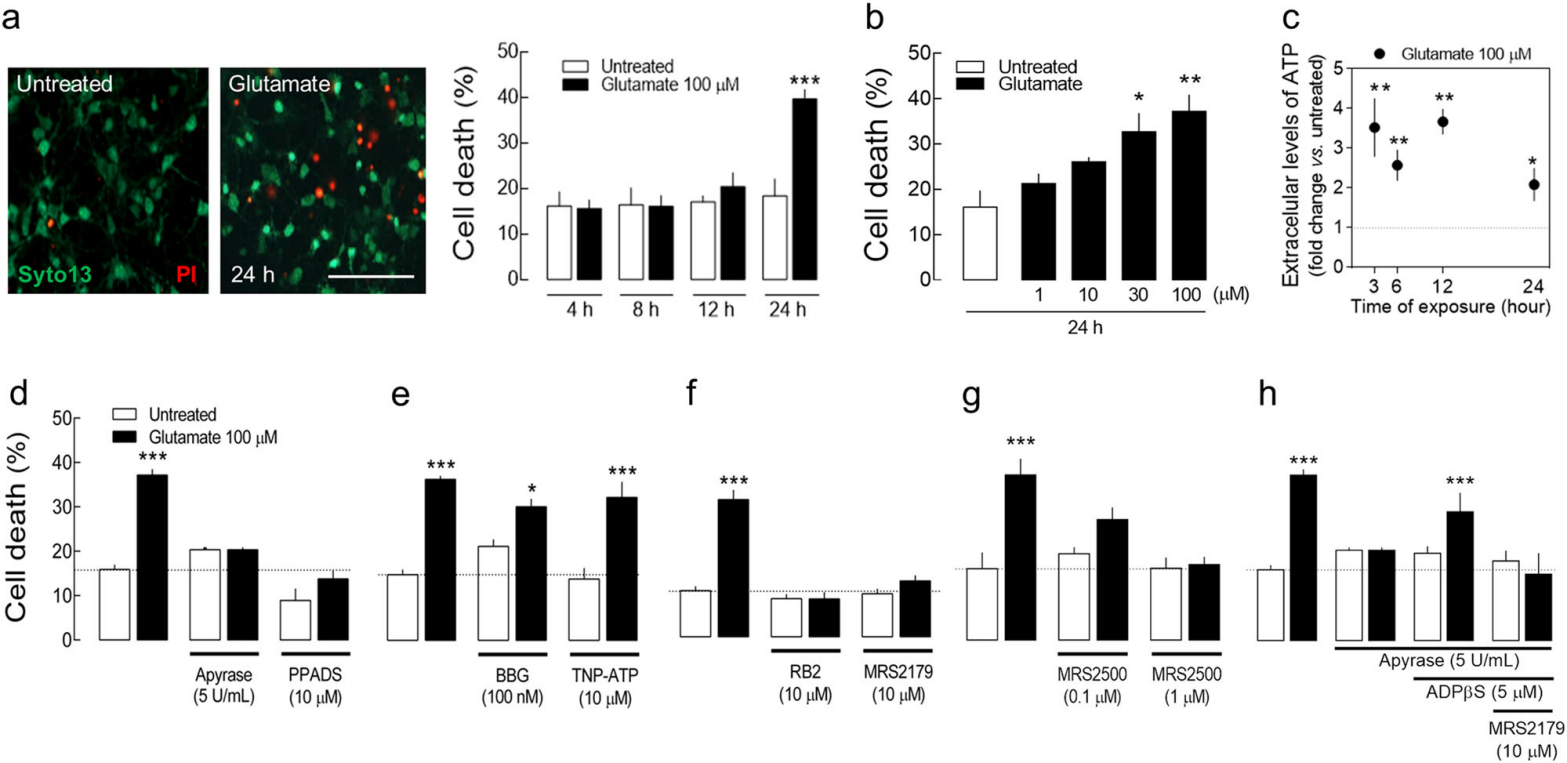
Extracellular ATP and P2Y1Rs are required for glutamate-induced neuronal death. **a** Rat hippocampal neurons were exposed to glutamate (100 µM) for 30 min, which induced a significant increase in cell death (PI—propidium iodide incorporation) 24 h later. The mean ± SEM percentage of dead cells was quantified from 8 to 12 cultures analyzing 300 cells per culture. Scale bar, 200 µm. ***p < 0.001 one-way ANOVA with Sidak’s test for time-matched comparisons. **b** Glutamate-induced neuronal death at 30 and 100 µM. *p < 0.05 and **p < 0.01 one-way ANOVA with Dunnet’s test vs. untreated. **c** Glutamate exposure induced a sustained increase of the extracellular levels of ATP. Data are expressed as the fold change in the levels of ATP in the culture media (mean ± SEM) vs. untreated; *p < 0.05 and **p < 0.01, one-sample t test (hypothetical value of 1). **d** Glutamate-induced neuronal death was not observed either in the presence of apyrase (5 U/mL) or PPADS (10 µM; P2R antagonist); **e** it persisted in the presence of Brilliant Blue G (BBG; 100 nM; P2X7R antagonist) or TNP-ATP (10 μM; P2X1-3-containing receptors antagonist); **f** was prevented by Reactive Blue 2 (RB2; 10 μM; P2YR-preferring antagonist), by MRS2179 (10 µM; P2Y1R antagonist); and **g** by MRS2500 (0.1 and 1 µM; P2Y1R antagonist). **h** In the presence of apyrase, ADPβS (5 µM; agonist of P2Y1,12,13Rs) restored glutamate-induced neuronal death, an effect prevented by MRS2179. Data are mean ± SEM percentage of dead cells quantified from 3 to 8 cultures analyzing 300 cells per culture. *p < 0.05 and ***p < 0.001 one-way ANOVA with Sidak’s test for glutamate vs. untreated

The exposure of rat hippocampal neurons to glutamate (30–100 μM) for 30 min induced a significant increase in neuronal death 24 h later (Fig. 1a, b). Glutamate (100 µM) triggered a sustained increase of the extracellular levels of ATP early on, prior to frank neuronal degeneration (Fig. 1c). The removal of extracellular ATP/ADP by apyrase (5 U/mL) was sufficient to abrogate glutamate-induced neuronal death (Fig. 1d). A similar neuroprotection was observed in the presence of the P2R antagonist, PPADS (Fig. 1d). Neither the selective antagonist of P2X7R, brilliant blue G, nor the antagonist of P2X1-3-containing receptors, TNP-ATP, mimicked this neuroprotection (Fig. 1e). Instead, the preferring P2YR antagonist, reactive blue 2, and the selective P2Y1R antagonist MRS2179 abrogated glutamate-induced neuronal death (Fig. 1f). Similar neuroprotection was observed with another P2Y1R antagonist, MRS2500, re-enforcing that the neuroprotection observed is due to the blockade of P2Y1R (Fig. 1g). This neuroprotection by P2Y1R blockade was observed in neurons both at 7 days in vitro (DIV7) and 14 days in vitro (DIV14). We further observed that in the presence of apyrase, glutamate neurotoxicity is restored by the addition of ADPβS (5 µM), an agonist of P2Y1, 12, 13 receptors, an effect prevented by the blockade of P2Y1R with MRS2179 (Fig. 1h). These results show that ATP/ADP through the activation of P2Y1R is involved in glutamate-induced neuronal death.

### Blockade of P2Y1R attenuates seizure-induced hippocampal neurodegeneration

**Fig. 2:**
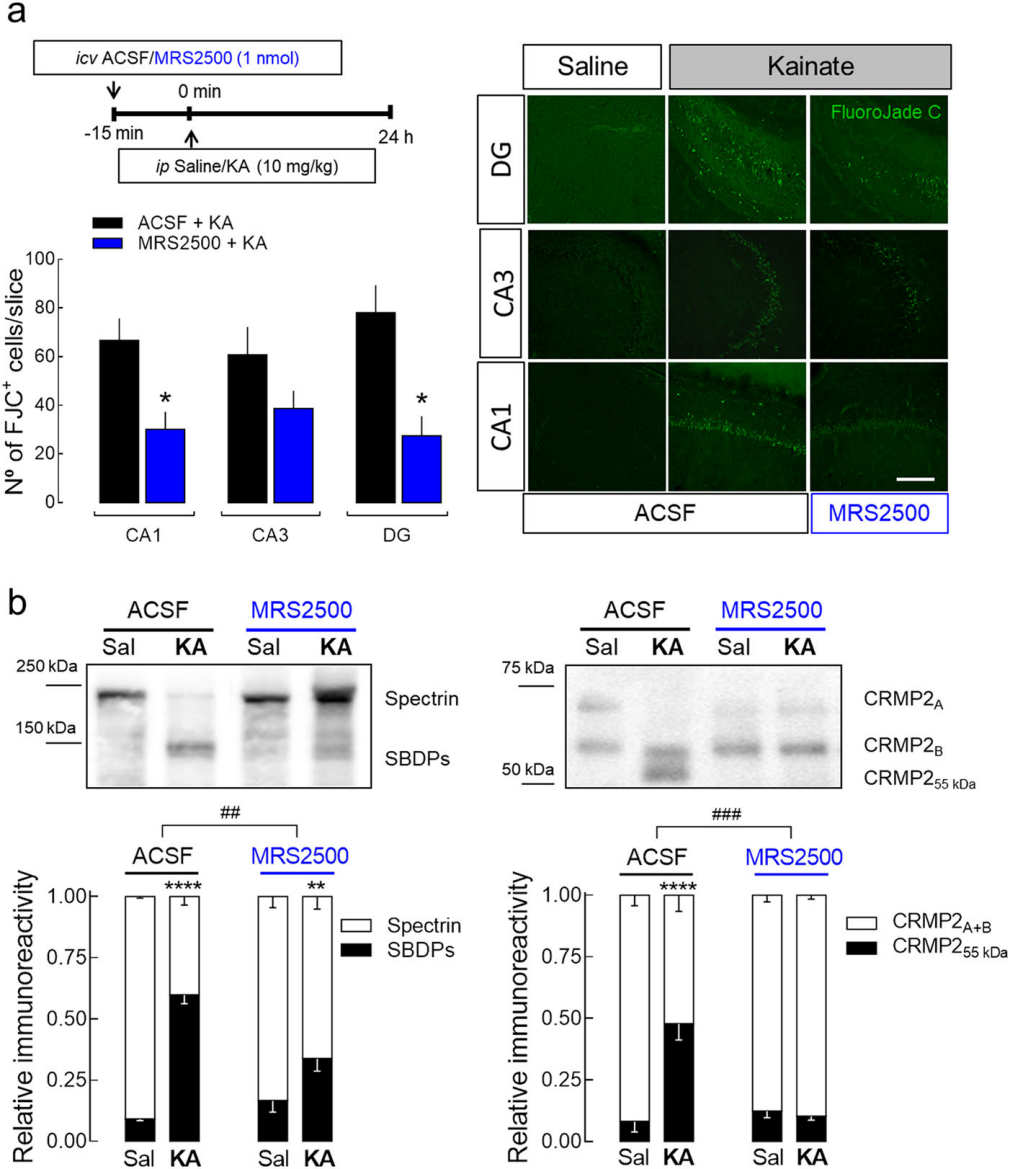
Pharmacological blockade of P2Y1Rs attenuates seizure-induced neurodegeneration in the rat hippocampus. **a** The intraperitoneal (i.p.) administration of 10 mg/kg of kainate (KA) in rats caused convulsive period of circa 2 h, which induced hippocampal neuronal death 24 h later, measured by FluoroJadeC-positive cells (FJC^+^), both in CA1, CA3, and dentate gyrus (DG) regions, as depicted in the representative images (scale bar, 200 µm) and quantified in the histogram. The hippocampi of animals injected with saline were absent of FJC^+^ cells. The number of FJC^+^ cells induced by KA was significantly attenuated by the prior i.c.v. injection of 1 nmol MRS2500 (15 min before KA injection), in DG and CA1, also showing a reduction tendency in CA3 region (*p* = 0.0815). The data are expressed as the number of FJC^+^ cells *per* slice (mean ± SEM) quantified from 4 animals *per* group and analyzing 12 coronal sections separated successively by 240 µm representing the entire hippocampus from each animal. *p < 0.05 one-way ANOVA with Sidaks’s test. **b** Western blot of calpain substrates spectrin and CRMP2 in hippocampal protein extracts prepared 24 h after saline/KA injection. KA-injected animals displayed an increase of the percentage of spectrin breakdown products (SBDPs) with an apparent molecular weight of ~145 kDa, and of the truncated form of CRMP2 (CRMP2_55_ kDa). The prior injection of MRS2500 attenuated or prevented the calpain-mediated cleavage of spectrin or CRMP2, respectively. Data are mean ± SEM of the relative percentage of Spectrin_full-length_/SBDPs and CRMP2_A+B_/CRMP2_55_ kDa (*n* = 4). **p < 0.01 and ****p < 0.0001 one-way ANOVA with Sidak’s test for KA vs. saline. ^##^*p* < 0.01 and ^###^*p* < 0.001 two-way ANOVA

To appraise if P2Y1R is involved in excitotoxicity in vivo, we used the systemic administration of kainic acid (KA), a widely used rat model of temporal lobe epilepsy, which is accompanied by hippocampal degeneration^25^ and used as an in vivo model of excitotoxicity^26^. Accordingly, neuronal loss (Fluoro-Jade C (FJC)-positive staining) both in CA1, CA3, and DG regions (Fig. 2a) was present 24 h after the intraperitoneal injection of KA, in animals that experienced at least stage III in the Racine’s scale^27^. A single intracerebroventricular (i.c.v.) injection of MRS2500 (1 nmol) 15 min prior to KA administration significantly decreased the number of FJC-positive cells in CA1 and DG, also displaying a decreasing tendency for the CA3 region (Fig. 2a). MRS2500 did not modify seizure behavior. These data show that P2Y1R antagonism is neuroprotective against excitotoxicity also in vivo.

We also observed an increased immunoreactivity of the 145 kDa spectrin breakdown product (SBDP) associated with calpain-mediated cleavage in the hippocampi from KA-injected rats (Fig. 2b), as previously reported^28^ and consistent with status-epilepticus-induced, calpain-mediated neuronal death^29^. Increased calpain activity was further confirmed by the observed cleavage of CRMP2 protein into CRMP2_55 kDa_ product, previously shown to be triggered under excitotoxic conditions in vitro^30^ (Fig. 2b). The administration of MRS2500 attenuated or prevented the calpain-mediated cleavage of spectrin or CRMP2, respectively (Fig. 2b). These findings not only confirm the involvement of P2Y1R in excitotoxicity-mediated neurodegeneration in vivo, but also provide a link between P2Y1R and calpain activity.

### P2Y1R is required for glutamate-induced and NMDAR-mediated rat hippocampal neuronal loss

**Fig. 3:**
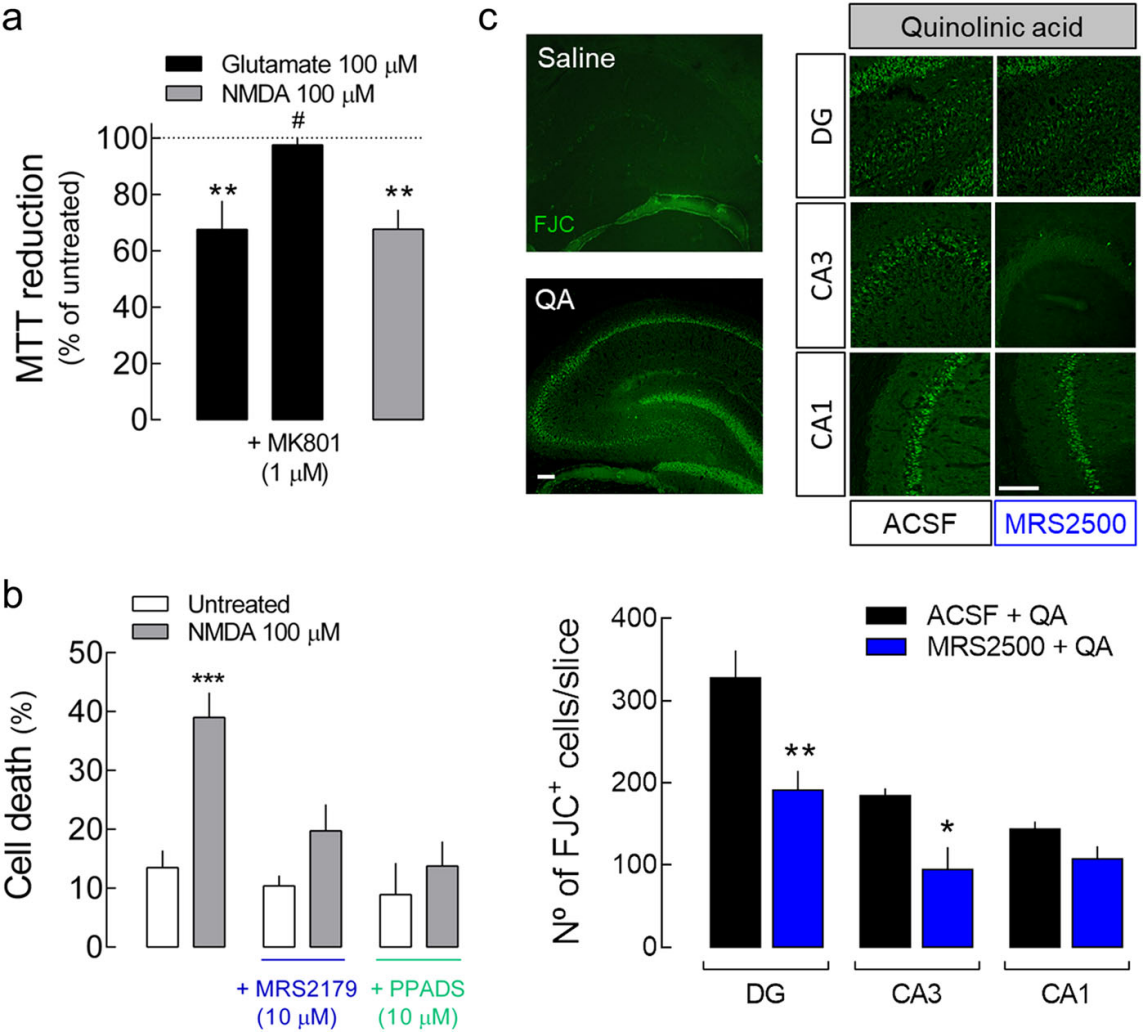
Glutamate induces cell death in rat hippocampal neurons through the activation of NMDARs in a P2Y1R-dependent manner. **a** The exposure of rat cultured hippocampal neurons to glutamate (100 µM) for 30 min induced a decrease in cell viability 24 h later, which was prevented by the NMDAR antagonist MK801 and mimicked by the exposure to NMDA (100 µM) for 30 min. Data are mean ± SEM of the percentage of MTT reduction vs. respective control, quantified from 4 to 7 different cultures, analyzing triplicates per condition per culture. **p < 0.01 one-sample *t* test (hypothetical value of 100); ^#^*p* < 0.05 unpaired t test for glutamate + MK801 vs. glutamate alone. **b** NMDA-induced neuronal death was prevented either by PPADS (10 µM) or MRS2179 (10 µM). The data are mean ± SEM of the percentage of dead cells (propidium iodide incorporation) quantified from four cultures analyzing 300 cells per condition per culture. ***p < 0.001 one-way ANOVA with Sidak’s test for NMDA vs. untreated. **c** The intrahippocampal administration of 120 nmol of quinolinic acid (QA) in rats caused hippocampal neuronal death 24 h later, measured as Fluoro-Jade C-positive cells (FJC^+^), as depicted in the representative images (scale bars, 200 µm) and quantified in the histogram. The co-administration of 1 nmol of MRS2500 significantly attenuated the number of FJC^+^ cells in DG and CA3 regions. The mean ± SEM number of FJC^+^ per slice was quantified from 4 animals per group and analyzing 12 coronal sections separated successively by 240 µm and representing the entire hippocampus from each animal. *p < 0.05 one-way ANOVA with Sidak’s test

Glutamate-induced hippocampal neuronal death was prevented by the NMDAR antagonist MK801 (Fig. 3a). Accordingly, a similar decrease of neuronal viability was observed when rat hippocampal neurons were exposed to NMDA (100 μM) instead of glutamate (Fig. 3a). These results confirm that glutamate-induced neuronal death is mediated by NMDARs, consistent with previous reports^4,5^. The NMDA-induced hippocampal neuronal death was prevented by P2Y1R antagonism (Fig. 3b).

Rat hippocampal neurodegeneration observed upon KA injection and attenuated by P2Y1R blockade (Fig. 2a) has been shown to be essentially dependent on NMDARs^31^. We further confirmed that P2Y1R antagonism affords neuroprotection against NMDAR-induced toxicity in vivo, by the observation of an attenuation of neuronal death induced by an intrahippocampal administration of the agonist of NMDARs quinolinic acid (QA, 120 nmol), with the co-injection of MRS2500 (Fig. 3c).

These results show that glutamate-induced neurotoxicity involves NMDARs and NMDAR-induced neuronal death also involves P2Y1R activation.

### The contribution of P2Y1R to glutamate-induced neuronal death fades with increasing severity of the excitotoxic stimuli

**Fig. 4:**
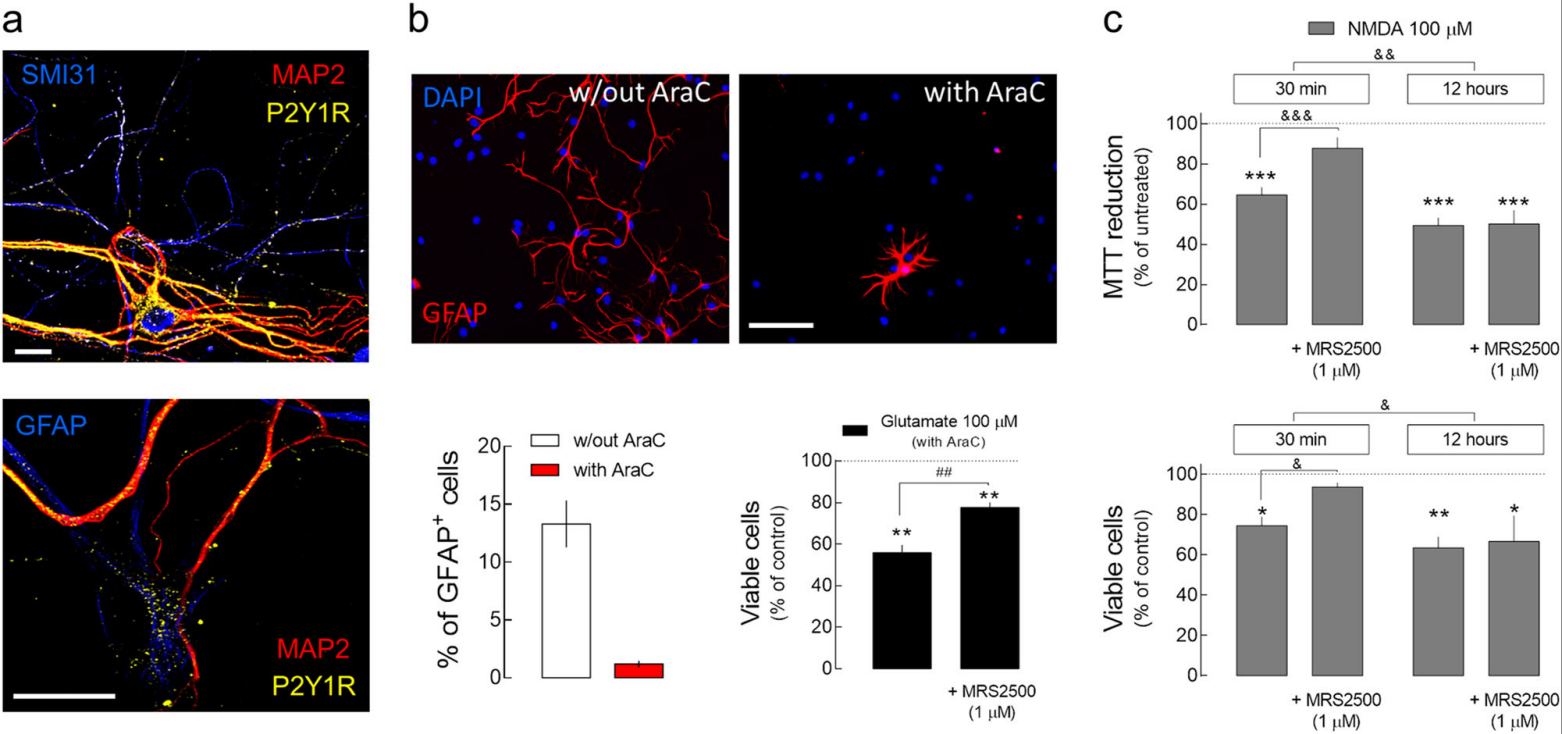
The contribution of P2Y1Rs to glutamate-induced neuronal death decreases with increasing severity of excitotoxic conditions. **a** Representative images of rat hippocampal primary cultures showing immunoreactivity for P2Y1Rs in neurons (MAP2—dendritic marker; SMI31—axonal marker) and astrocytes (GFAP^+^). Scale bars, 20 µm. **b** Primary hippocampal cultures incubated with AraC (2 µM) at DIV2–5 to inhibit astrocyte proliferation presented a reduced number of GFAP^+^ cells (DIV7), as depicted in the representative images (scale bar, 50 µm) and quantified in the left histogram. In cultures incubated with AraC, the exposure to glutamate (100 µM) for 30 min induced a significant increase in neuronal death (PI incorporation) observed 24 h later, which was attenuated by the presence of MRS2500 (1 µM). Data are mean ± SEM percentage of viable cells vs. respective control, quantified from four different cultures analyzing 300 cells per culture per condition. **c** The exposure of rat cultured hippocampal neurons to NMDA (100 µM) for 30 min induced a decrease in cell viability 24 h later evaluated both by MTT reduction (upper graph) and PI incorporation (lower graph), which was not observed in the presence of MRS2500 (1 µM). The decrease in cell viability observed 24 h after the exposure to NMDA (100 µM) for 12 h was not modified by MRS2500. Upper graph—data are mean ± SEM of the percentage of MTT reduction vs. respective control, quantified from 5–8 different cultures, analyzing triplicates per condition per culture. Lower graph—data are mean ± SEM of the percentage of dead cells (PI incorporation) vs. respective control quantified from four cultures analyzing 300 cells per condition per culture. *p < 0.05, **p < 0.01, and ***p < 0.001 one-sample t test (hypothetical value of 100); ^##^*p* < 0.01 unpaired *t* test; ^&^*p* < 0.05, ^&&^*p* < 0.01, and ^&&&^*p* < 0.001 two-way ANOVA with Sidak’s multicomparison test

P2Y1Rs are expressed both in rat hippocampal neurons and astrocytes (e.g. Rodrigues et al.^18^ and Bowser and Khakh^32^). Accordingly, we could detect immunoreactivity for P2Y1R both in cultured neurons and astrocytes (Fig. 4a). Therefore, we evaluated the putative contribution of astrocytic P2Y1R to glutamate-induced neurotoxicity. Despite the fact that we observed a neuroprotection by P2Y1R blockade not only in DIV14 but also in DIV7, where the percentage of astrocytes is relatively low (<15%), we evaluated the contribution of P2Y1R to glutamate-induced neuronal death now in cultures exposed to cytosine 1-β-d-arabinofuranoside (AraC) to inhibit astrocyte proliferation (Fig. 4b). In these conditions, we could observe that the blockade of P2Y1R with MRS2500 only attenuated glutamate-induced neuronal death. This shows that neuronal P2Y1Rs contribute to glutamate-induced neurotoxicity. But it also suggests for a contribution of astrocytic P2Y1Rs. However, the neuronal death caused by glutamate exposure was considerably higher in cultures in which astrocyte proliferation was inhibited (~45% neuronal death; Fig. 4b) in comparison with regular primary cultures (20–25%), most likely due to the lack of glutamate uptake by astrocytes. This also raises the possibility that the contribution of P2Y1R may be reduced with more intense excitotoxic conditions. Accordingly, while the presence of MRS2500 prevented the decrease of cell viability observed 24 h after the exposure to NMDA (100 µM) for 30 min, it failed to modify the neuronal death caused by the exposure to NMDA (100 µM) for 12 h (Fig. 4c).

These results show that neuronal P2Y1Rs contribute to glutamate-induced neuronal death, and also indicate that this contribution fades with more intense excitotoxic conditions.

### P2Y1R induces an increase in NMDAR-mediated Ca^2+^ entry selectively at the axonal compartments

**Fig. 5:**
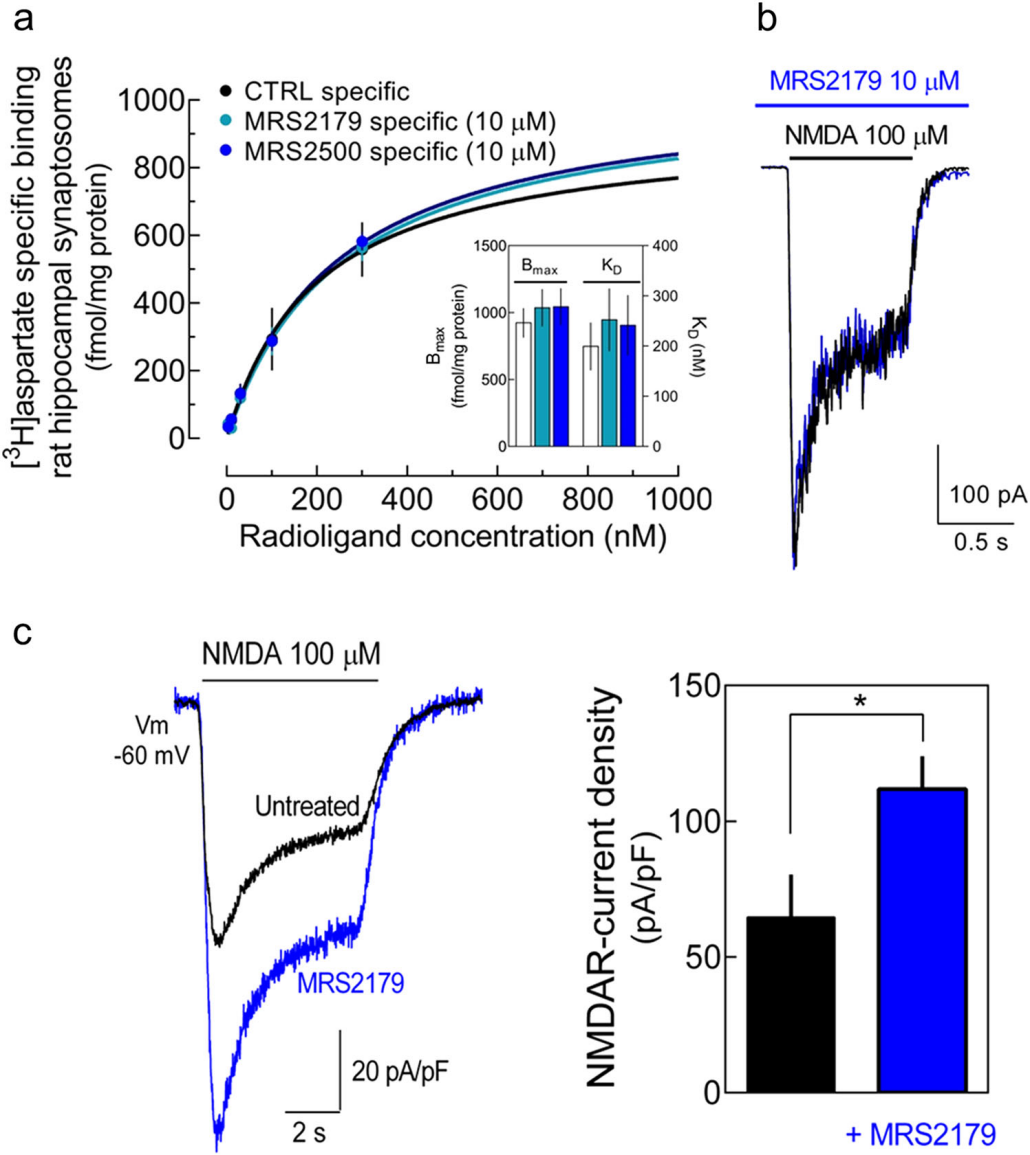
Pharmacological blockade of P2Y1Rs increases NMDAR-induced current density in rat hippocampal neurons. **a** [^3^H]Aspartic acid saturation-specific binding curve to NMDARs in rat hippocampal synaptosomal membranes with a *B*_max_ of 923.9 ± 110.5 fmol/mg protein and with a *K*_D_ of 199 ± 48 nM (mean ± SEM; *n* = 7). The non-NMDAR binding was defined in the presence of D-AP-5 (30 µM). Neither MRS2179 (10 µM) nor MRS2500 (10 µM) affected the *B*_max_ and the *K*_D_ values of [^3^H]aspartic acid specific binding (bar graphs; *n* = 6). **b** Representative traces of NMDA-induced inward currents in rat hippocampal neurons in the absence and in the presence of MRS2179 (10 µM). **c** Rat hippocampal neurons exposed to MRS2179 (10 µM) for 30 min presented a higher NMDA-induced current density in relation to untreated cells. Data are expressed as the mean ± SEM of peak NMDA-induced current density (pA/pF). *p < 0.05 unpaired *t* test (*n* = 6–7)

Neither MRS2500 nor MRS2179 affected the ligand binding to NMDARs (Fig. 5a) or modified NMDARs activation and current (Fig. 5b). This discarded any eventual off-target action of the P2Y1R antagonists tested directly on NMDARs, which could be affording the neuroprotection observed. Hence, we hypothesized that P2Y1R could be contributing to glutamate-induced neurodegeneration by inducing an increase in the density of NMDARs and consequently favoring a toxic NMDAR-mediated Ca^2+^ entry. Surprisingly, rat hippocampal neurons incubated for 30 min with MRS2179 displayed a higher NMDAR-induced current density in comparison with untreated neurons (Fig. 5c), which is at odds with the neuroprotection afforded by the P2Y1Rs antagonism against NMDARs toxicity.

**Fig. 6:**
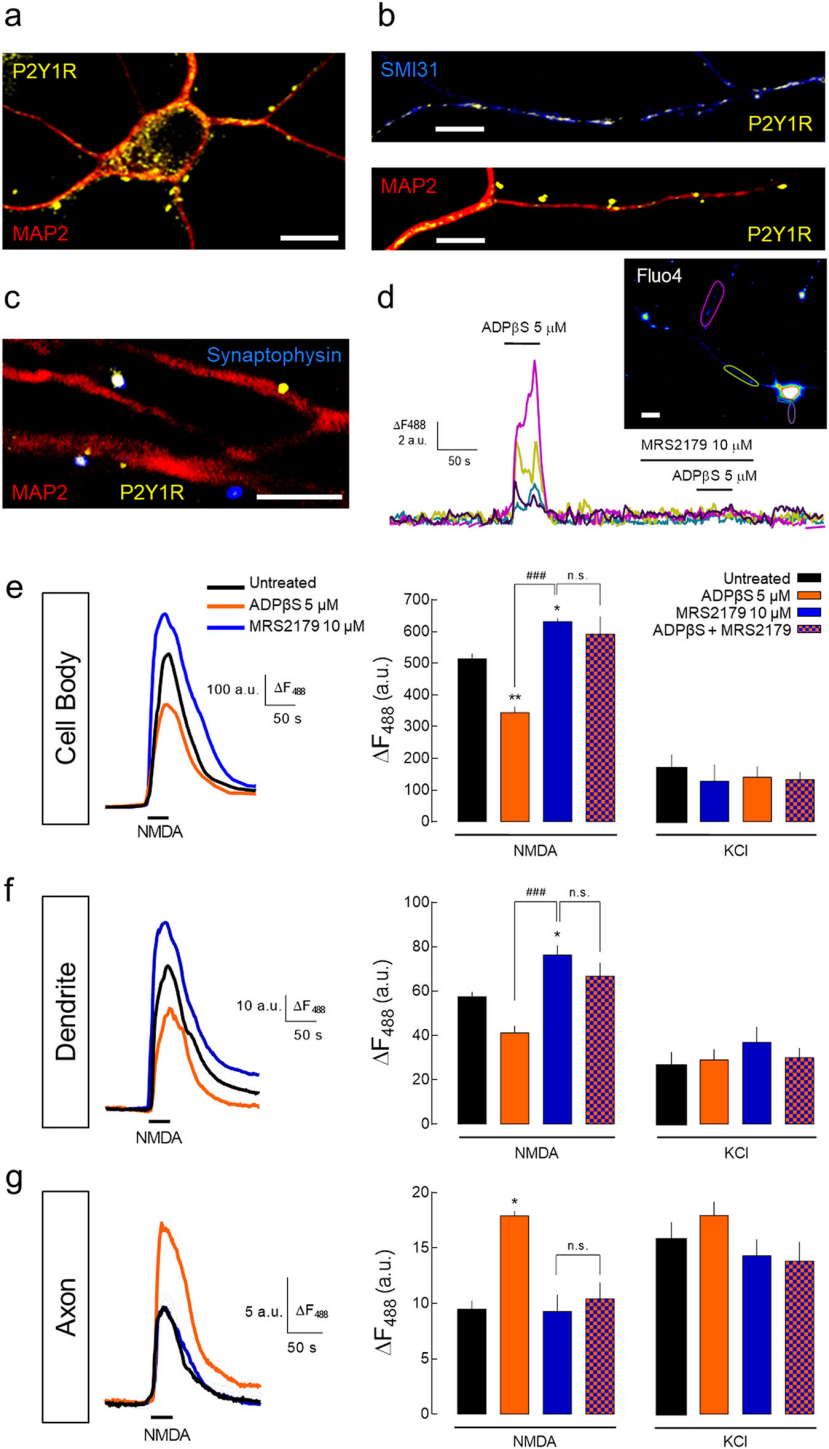
P2Y1Rs differentially regulate NMDARs in a subcellular-specific manner. **a–c** Representative images of rat hippocampal neurons showing the immunoreactivity for P2Y1Rs in **a** the cell body, **b** dendrites (MAP2—dendritic marker), predominantly in proximal regions, axons (SMI31—axonal marker), and **c** nerve terminals (synaptophysin—synaptic marker). **d** Representative traces of Ca^2+^-imaging analysis in rat hippocampal neurons (loaded with Fluo-4) showing [Ca^2+^]_i_ transients represented by fluorescence increase (Δ*F*_488_) induced by ADPβS (5 µM) and abrogated by MRS2179 (10 µM), recorded in dendrites (purple), soma (blue), and in proximal and distal axon (yellow-green and magenta, respectively). Scale bars, 10 µm. **e–g** Ca^2+^ imaging of rat hippocampal neurons previously exposed to untreated conditions, ADPβS (5 µM), MRS2179 (10 µM), or ADPβS + MRS2179 for 30 min, and challenged with NMDA (100 µM) and KCl (30 mM). In neurons previously exposed to ADPβS, the perfusion of NMDA for 1 min induced lower Ca^2+^ transients in **e** the soma and **f** dendrites, and a higher Ca^2+^ rise in **g** axons in relation to untreated cells, as depicted in the average traces of fluorescence intensity and quantitatively summarized in the histograms. These ADPβS-induced effects were not observed in the presence of MRS2179, which per se induced a higher NMDA-induced Ca^2+^ transients in the soma and dendrites, but not in axons. The chemical depolarization with KCl induced similar Ca^2+^ transients in the neurons from each condition. The mean ± SEM of Δ*F*_488_ was quantified from four different cultures and analyzing a minimum of eight cells per culture per condition. *p < 0.05 and **p < 0.01 vs. untreated and ^###^*p* < 0.001 between indicated bars, one-way ANOVA with Sidak’s test. NS nonsignificant

As whole-cell patch-clamp recordings essentially provide total cell current, this analysis could be masking any eventual subcellular-specific P2Y1R-driven increase in NMDARs. To test this hypothesis, we first evaluated the subcellular distribution of P2Y1Rs in rat hippocampal neurons. P2Y1R immunoreactivity was found in the soma (Fig. 6a), dendrites, and axons (Fig. 6b), as similarly described in mice hippocampal neurons^33^. The co-localization of P2Y1R with synaptophysin also indicated the presence of P2Y1Rs in some synaptic contacts (~20% of synaptophysin puncta; Fig. 6c). This subcellular localization of P2Y1Rs was functionally confirmed by Ca^2+^ imaging. ADPβS-induced Ca^2+^ rises both in the soma and dendrites, and predominantly in distal axons, both absent in the presence of MRS2179 (Fig. 6d).

Using Ca^2+^-imaging analysis, neurons pre-incubated with MRS2179 for 30 min displayed a higher NMDA-induced Ca^2+^ transients both in the soma and dendrites (Fig. 6e, f), similar to the observed in patch-clamp recordings (Fig. 5c). Accordingly, the cells pre-incubated with ADPβS presented a lower NMDA-induced Ca^2+^-transients in the soma and dendrites, an effect prevented by MRS2179 (Fig. 6e, f). In contrast, the cells incubated with ADPβS presented ~2-fold higher NMDA-induced Ca^2+^ transients in axonal compartments (Fig. 6g), an effect prevented by MRS2179 (Fig. 6g). MRS2179 per se did not modify NMDA-induced Ca^2+^ transients in axons. Ca^2+^ transients triggered by the chemical depolarization with KCl (30 mM) were not significantly modified by the pharmacological manipulation of P2Y1Rs in any of the cellular compartments (Fig. 6e–g) discarding the involvement of a P2Y1R-driven regulation of voltage-gated calcium channels (VGCCs). Hence, these results show that P2Y1R modulates in a bidirectional and subcellular-specific manner the NMDA-induced Ca^2+^ entry, decreasing it in the soma and dendrites and increasing it in the axons.

### Blockade of P2Y1R prevents glutamate-induced initial axonal cytoskeleton damage

**Fig. 7:**
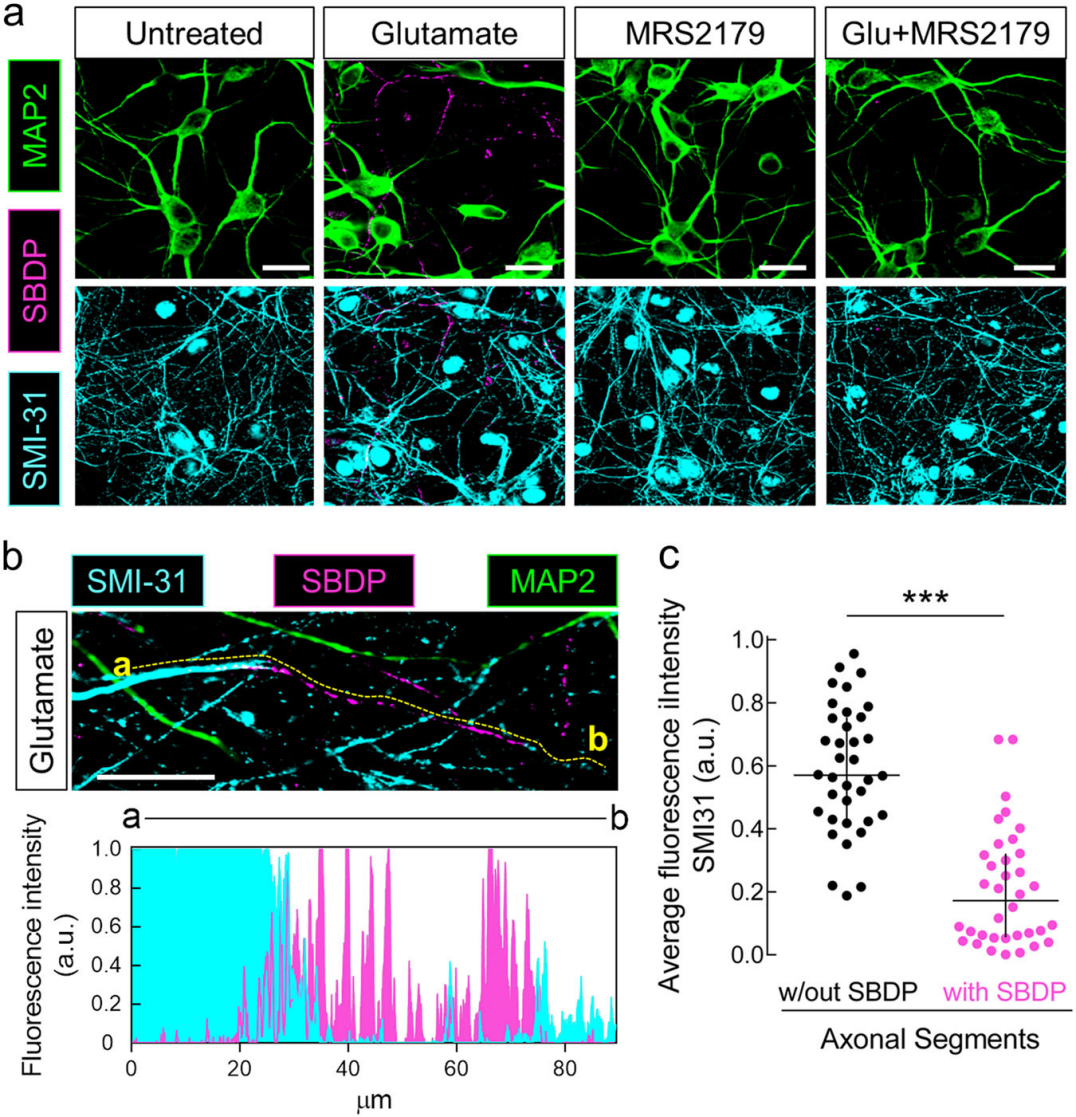
Glutamate induces an initial calpain activation and cytoskeleton degeneration in the axon of rat hippocampal neurons in a P2Y1R-dependent manner. **a** Representative images of immunocytochemical analyses of rat hippocampal neurons (DIV14) showing spectrin breakdown products (SBDPs—magenta), 1 h after glutamate exposure (100 µM; 30 min) in axons (SMI31; axonal marker; cyan), but not in dendrites (MAP2; dendritic marker; green), which was not observed in the presence of the selective antagonist of P2Y1Rs, MRS2179 (10 µM). **b** Representative image depicting a coincident reduction of SMI31 immunoreactivity (p-neurofilaments) with the labeling of SBDPs in the axonal structure defined by the yellow dashed line between points a and b, as quantitatively represented in the lower graph. **c** In the neurons exposed to glutamate, the axonal structures immunopositive for SBDPs present a reduced average in the fluorescence intensity of SMI31 in comparison to axonal segments negative for SBDPs. Data are expressed as the median with interquartile range of the average fluorescence intensity of SMI31 quantified from 36 segments with a minimum length of 50 µm from axonal structures without or with SBDP immunoreactivity. Dots represent single segments. ***p < 0.001 Mann–Whitney test. In **b**, **c**, the fluorescence intensities from the 8-bit images (256-color scale) were directly converted into a 0–1 scale, being the absence of fluorescence set as 0 and the maximum intensity set as 1. Scale bars, 20 µm

Neuritic degeneration caused by excitotoxicity precedes cell soma demise and involves calpain activity^34^. Calpain-mediated neuronal injury is associated with NMDAR-mediated Ca^2+^ overload^4,35^. Thus, the identification of a P2Y1R-driven increase in NMDAR-mediated Ca^2+^ entry selectively at the axons prompted the hypothesis that P2Y1Rs may be contributing to neurodegeneration by promoting a toxic increase in axonal NMDAR-mediated Ca^2+^ entry and subsequent calpain-mediated axonal cytoskeleton damage. Indeed, in rat hippocampal neurons, we detected immunoreactivity with an antibody that binds specifically the product resulting from the cleavage of spectrin by calpain I^36^, 1 h after the exposure to glutamate (Fig. 7a). This is in agreement with the previous observation of fast calpain activation, peaking 30 min after the excitotoxic insult^37^. Surprisingly, this immunoreactivity was solely observed in structures immunostained with SMI31, which reacts predominantly with phosphorylated neurofilament H present in axons, but absent in MAP2-positive structures (dendrites). Furthermore, we could observe a decreased SMI31 immunoreactivity in the segments immunopositive for SBDPs (Fig. 7b, c). This shows a match between calpain activity and axonal cytoskeleton damage, particularly showing the degradation of neurofilaments, which are calpain substrates^38^. The glutamate-induced immunoreactivity for SBDPs was not observed in the presence of MRS2179 (Fig. 7a). These data show that glutamate induces calpain activation and cytoskeletal damage initially in axons in a P2Y1R-dependent manner.

### P2Y1 receptor activation contributes for glutamate-induced early synaptic loss

**Fig. 8:**
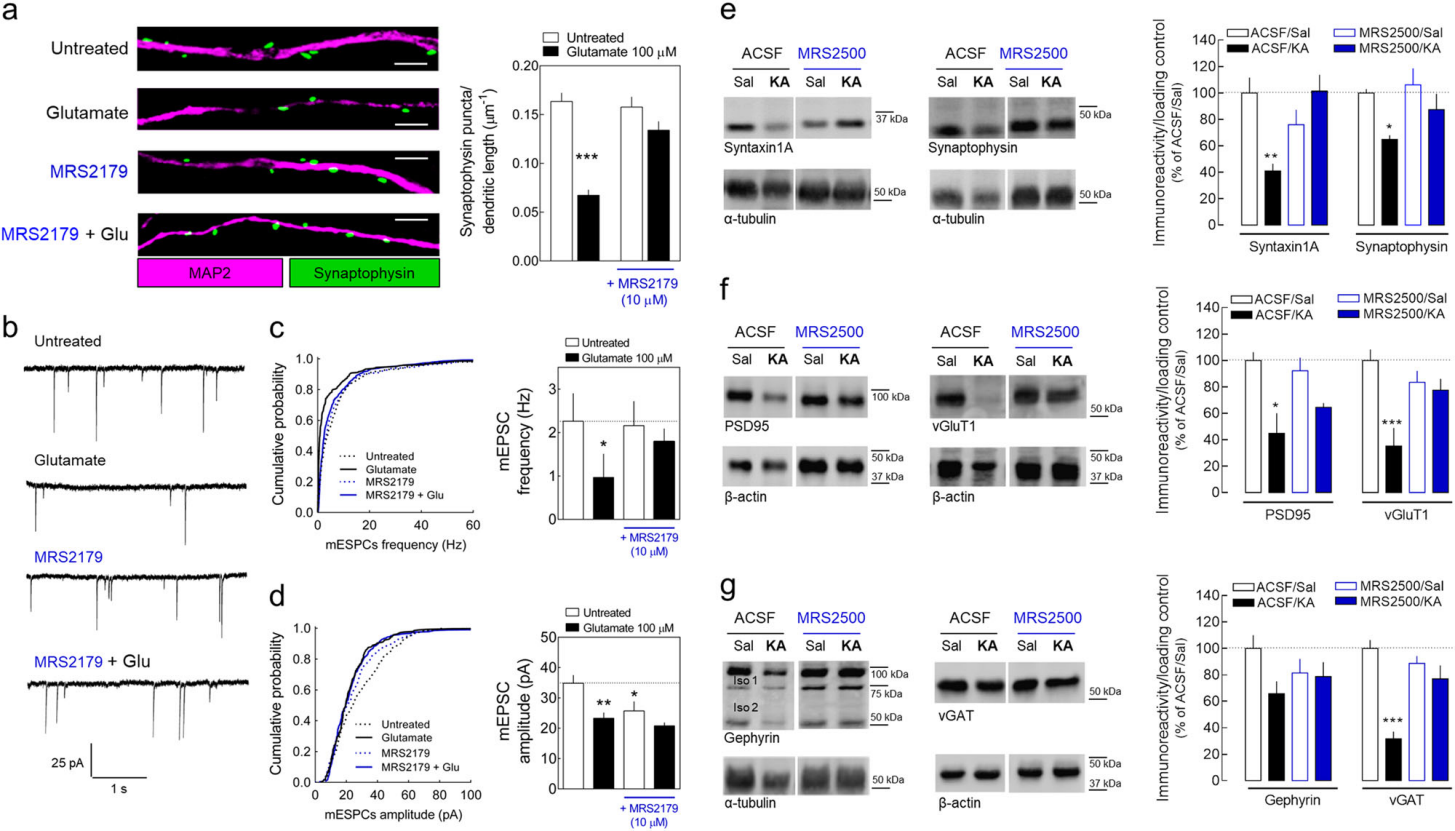
Glutamate induces synaptic loss prior to neuronal death in a P2Y1R-dependent manner. **a** Rat hippocampal neurons (DIV14) exposed to glutamate (100 µM; 30 min) display a decrease in the density of the synaptophysin puncta per dendritic length after 12 h, an effect not observed in the presence of MRS2179 (10 µM), as depicted in the representative confocal images (scale bar, 10 µm). Data are mean ± SEM of synaptophysin puncta per dendritic length quantified from four different cultures analyzing a minimum of 2000 µm of MAP2^+^ structures from two independent coverslips per condition, per culture. **b** Representative traces of mEPSC recorded in rat hippocampal neurons (DIV14) 12–14 h after the exposure to glutamate (100 µM; 30 min) either in the absence or in the presence of MRS2179 (10 µM). **c**, **d** Cumulative histograms (left) and pooled data (right) of the average of **c** instantaneous frequency or **d** peak amplitude of mEPSCs. Pooled data are mean ± SEM of the average frequency/amplitude of mEPSCs calculated from 180 s bins recorded from 11 to 15 neurons per condition. *p < 0.05 and **p < 0.01 Kruskal–Wallis with Dunn’s test for frequency and one-way ANOVA with Sidak’s test for amplitude. **e** Western blot analysis of general synaptic markers, synaptophysin and syntaxin1A, **f** glutamatergic synaptic markers, vGluT1 and PSD-95 and **g** GABAergic synaptic markers, gephyrin and vGAT, in total protein extracts prepared from hippocampi of rats previously injected i.p. with saline or kainate (KA,10 mg/kg). Hippocampal protein extracts of the animals injected with KA displayed a significant decrease in the immunoreactivity of all the synaptic proteins evaluated, except for gephyrin (*p* = 0.1205), in comparison with saline-injected mice, as depicted in the representative Western blots (in left) and summarized in the histograms (in right). The i.c.v. injection of 1 nmol of MRS2500 15 min prior to the administration of KA prevented or attenuated the decrease in the immunoreactivity of the different synaptic markers. MRS2500 per se did not cause any significant modification in the density of the synaptic markers. Data are percentage (mean ± SEM) of the ratio of immunoreactivity synaptic markers/loading control relative to the average ratio obtained in samples from aCSF/Sal group (*n* = 4). *p < 0.05, **p < 0.01, and ***p < 0.001 one-way ANOVA with Sidak’s post hoc test for KA vs. respective saline

Synaptic dysfunction/loss is a primordial step in the cascade of neurodegenerative events in different brain disorders^39^. Accordingly, we observed a significant reduction of the synaptic density in rat hippocampal neurons 12 h after glutamate exposure gauged both by morphological analysis (Fig. 8a) and by a reduction of the frequency of miniature excitatory post-synaptic currents (mEPSCs; Fig. 8b, c). These data demonstrate a synaptotoxicity induced by glutamate prior to frank neuronal degeneration, only significant 24 h later (Fig. 1a). This glutamate-induced synaptic loss also involves P2Y1R since it was prevented by the presence of MRS2179 (Fig. 8a–c). We also observed a decrease in the amplitude of mEPSCs in neurons exposed to glutamate, which was also P2Y1R-dependent (Fig. 8d).

A decrease of the immunoreactivity of synaptic markers was also observed in the rat hippocampus 24 h upon KA injection. A significant reduction of the immunoreactivity of general synaptic markers sintaxin1A and synaptophysin (Fig. 8e), the glutamatergic synaptic markers vGluT1 and PSD-95 (Fig. 8f), and the GABAergic synaptic markers vGAT and gephyrin (Fig. 8g) was appraised. The single i.c.v. injection of MRS2500 15 min prior to KA administration prevented/attenuated this seizure-induced decrease in the immunoreactivity of the synaptic markers (Fig. 8e–g). These data show the existence of a synaptic loss induced by excitotoxicity prior to neuronal death, which also involves P2Y1R activation.

## Discussion

In the present study, we demonstrate both in vitro and in vivo that excitotoxicity entails extracellular ATP/ADP and P2Y1R activation. We found that glutamate triggers a sustained increase of extracellular ATP levels, which contributes for glutamate-induced hippocampal neuronal death through the activation of P2Y1R. This contribution of P2Y1Rs fades with increasing intensity of excitotoxic conditions. This indicates that P2Y1R is not contributing directly to neurodegeneration, rather behaving as a catalyst of excitotoxicity by decreasing the threshold from which glutamate becomes neurotoxic. Moreover, we found that excitotoxicity began with an early synaptotoxicity that was also prevented/attenuated by the antagonism of P2Y1R, both in vitro and in vivo. This should be due to a P2Y1R-driven subcellular-specific gain-of-function of NMDARs, favoring an increase to toxic levels of NMDAR-mediated Ca^2+^ entry, leading to calpain-mediated axonal cytoskeleton damage.

Several evidences support the involvement of extracellular ATP and P2Rs in the pathogenesis of a wide range of brain disorders. Glutamate-mediated excitotoxicity is a pathogenic event associated with different acute and chronic brain disorders^2^. Thus, the present data re-enforces a prominent role of ATPergic signaling in brain pathological conditions. Besides, although a major focus has been dedicated to P2X7R probably acting through the control of neuroinflammation^13^, our data pinpoints a particular pathological role of P2Y1Rs.

P2Y1Rs have been shown to be involved in neurodegeneration in ischemic conditions^14^ and trauma^16^. The neuroprotection afforded by P2Y1R blockade in those pathological conditions has been attributed to the control of astrocytic function^14,16,17^. On the other hand, P2Y1Rs are expressed in neurons^18,32^ and it has been shown that neuronal P2Y1R may also directly affect brain damage^15^. In the present study, in low-astrocyte cultures, we could still observe neuroprotection afforded by P2Y1R blockade, supporting the involvement of neuronal P2Y1Rs (Fig. 4b). The observation of a partial neuroprotection by the antagonism of P2Y1Rs in these cultures could also indicate for an additional contribution of astrocytic P2Y1Rs. The ability of P2Y1Rs to induce glutamate release from astrocytes^40^, subsequently activating NMDAR on neurons^41^ could be a mechanism through which astrocytic P2Y1Rs could be promoting neuronal death. Yet, ADPβS is not neurotoxic per se. Besides, in such scenario, the removal of astrocytes would be expected to have a pro-survival action. Instead, we observed a higher cell death. This is most likely due to the lack of glutamate uptake by astrocytes, which could mask a pro-survival effect arising from the removal of neurotoxic astrocytic P2Y1Rs. However, we also observed that the blockade of P2Y1Rs failed to modify the neuronal death caused by a more prolonged excitotoxic stimulus (Fig. 4c) showing a decreased impact of P2Y1Rs with more intense excitotoxic conditions. Thus, although not fully discarding a partial contribution of the P2Y1Rs expressed in astrocytes, the available data favors a prominent involvement of neuronal P2Y1Rs, also indicating that P2Y1Rs are not directly neurotoxic, rather behaving as promoters of the conditions that favor a neurotoxic action of glutamate.

The contribution of P2Y1Rs as promoters rather than effectors in excitotoxicity is more in agreement with the observation of attenuation rather than a prevention of the seizure-induced or NMDAR-mediated hippocampal degeneration by the blockade of P2Y1Rs (Figs. 2a and 3c). Besides, it also agrees with the different neuroprotective profiles displayed by P2Y1R antagonism in each model, attenuating neuronal death in DG and CA3 regions in QA model (Fig. 3c), whereas in the KA model it afforded neuroprotection in DG and CA1, displaying a lower effect in CA3 (Fig. 2a). This may not be due to a region-specific neuroprotective action of the blockade of P2Y1Rs, as it was neuroprotective in CA3 in the QA model and in CA1 in the KA model. Instead, it may be due to the well-known selective vulnerability of hippocampal neurons to different excitotoxins, related with the distribution of the different ionotropic glutamate receptors across the different hippocampal regions^42^. In the QA model, NMDAR-mediated neurodegeneration is triggered by direct activation of NMDARs, whereas in the KA model NMDAR-mediated neuronal death is elicited indirectly through the increased neuronal excitability driven by AMPA and KA receptors^31^. For instance, KA administration induces hippocampal damage primarily in CA3^43^, a region particularly enriched with KARs^44^. As the contribution of P2Y1Rs fades with increasing excitotoxic stimulus, the neuroprotection afforded by P2Y1R antagonism is expected to be reduced/absent in regions where the excitotoxic stimulus is more severe, as observed in the CA3 region in the KA model.

Glutamate-induced neurotoxicity is well associated with intracellular Ca^2+^ rise^4^, mostly associated with the Ca^2+^ influx through NMDARs rather than non-NMDARs or VGCCs^45^. Consistently, we observed that glutamate-induced neurotoxicity was prevented by NMDAR blockade and mimicked by NMDAR agonism, which was also dependent on P2Y1R activation (Fig. 3). Furthermore, cell death has been mainly associated with the activation of extrasynaptic NMDARs^46^. Yet, this notion of toxic extrasynaptic NMDARs vs. pro-survival synaptic NMDARs is under debate^47^ and may essentially depend on the Ca^2+^ load mediated by NMDARs^48^. Regardless the involvement of extrasynaptic and/or synaptic NMDARs, the excessive activation of NMDARs has been indicated to primarily damage post-synaptic structures. However, the present results clearly point towards an initial degeneration of axonal compartments upon the exposure to glutamate before any detectable dendritic damage, raising axonal degeneration as a primary degenerative event in excitotoxicity. Such glutamate-induced axonal damage seems to be triggered by a P2Y1R-driven increased axonal NMDARs since: (i) P2Y1Rs are predominantly located at the axons (Fig. 6b, d); (ii) glutamate-induced axonal cytoskeleton damage depends on P2Y1R activation (Fig. 7a); (iii) P2Y1Rs increase NMDA-induced Ca^2+^ transients selectively at the axons (Fig. 6e–g); (iv) this should be due to a P2Y1R-driven increase in axonal NMDARs instead of a regulation of VGCCs. This view agrees with previous evidence showing that the distal axons are endowed with NMDARs^49^ and can be a direct target for excitotoxicity^50^. In addition, the results obtained further indicate that this increase of the axonal NMDARs should only occur in pathological conditions as P2Y1R blockade modified NMDA-induced Ca^2+^ transients in dendrites and soma but not in axons. Only the pharmacological activation of P2Y1Rs modified axonal NMDARs. Thus, in contrast to dendrites and soma, where there is a constitutive tonic regulation of NMDARs by P2Y1Rs, as previously observed in other preparations^20^, the P2Y1R-driven increase of axonal NMDARs should only occur in pathological conditions, most likely supported by a sustained increase of the extracellular ATP levels (Fig. 1b). Perhaps more surprisingly is the fact that the NMDAR-mediated Ca^2+^ transients in axons are much smaller in comparison to the observed both in dendrites and soma. This suggests that the toxic action of NMDAR may not be determined by the amount of Ca^2+^ entry. Instead, it may essentially depend on the balance between the Ca^2+^ load and the buffering capacity of the cell to preserve Ca^2+^ homeostasis, and/or on a more efficient coupling to degenerative pathways.

Although there is not a clear evidence demonstrating a causal relationship between axonal damage and synaptic loss, axon degeneration is a phenomenon shared by both acute and chronic neurodegenerative diseases^51–53^, which are characterized by an initial synaptic dysfunction/loss and neuritic degeneration prior to soma demise^39,54^. Preventing axonal damage and synaptic loss rather than protecting cell bodies has been proposed as the most effective therapeutic strategy to prevent neurodegeneration^52^. This is heralded by the observation in motor neuron disease models that protecting cell bodies from death has no impact on disease progression^55^, whereas the inhibition of axon degeneration and synaptic loss attenuated neuronal apoptosis^56^. Thus, although the degeneration of neuronal processes is mechanistically independent of cell death pathways, not involving caspase^57,58^, but rather calpain^34,38^, as also supported by the present data, the initial degeneration of axon seems not only to precede but also to constitute a trigger of apoptotic neuronal death. Accordingly, we observed that MRS2179 applied 12 h after glutamate exposure, when synaptic loss is evident in the absence of neuronal death, did not prevent glutamate-induced cell death. Altogether, these evidences point out the prevention of axonal damage as the most promising therapeutic strategy to arrest brain disorders^53^. We have now determined that axonal degeneration, at least in excitotoxic conditions, is favored by P2Y1R by promoting calpain-mediated axonal cytoskeleton damage, most likely reflecting a P2Y1R-driven increase of axonal NMDAR-mediated Ca^2+^ entry. Interestingly, calpains are also associated with the pathogenesis of different brain diseases^10^. Thus, this novel pathway may constitute a degenerative mechanism shared by different brain diseases, particularly relevant at initial pathogenic stages.

The use of NMDAR antagonists as therapeutic strategies cannot be exploited due to the vital roles of NMDARs^59^. Also, the lack of tools to selectively manipulate the downstream calpain activity precluded targeting these proteases as a therapeutic strategy^10^. Our findings now identify P2Y1Rs as a novel upstream potential therapeutic target and prompt to consider the use of P2Y1R antagonists as a promising strategy for therapeutic intervention in neurodegenerative diseases, at least to delay their onset/manifestation.

## Materials and methods

### Animals

Male Wistar rats (250–300 g; 8–10 weeks) were group housed, maintained on a 12 h light/dark cycle, and allowed food and water ad libitum. For the preparation of primary neuronal cultures, male and female rat Wistar embryos (E18) were used. All the procedures relating to the handling and killing of animals were approved and supervised by the Animal Care and Use Committee of the Center for Neuroscience and Cell Biology, Portugal, and of the *Instituto de Neurociencias*, CSIC-UMH, Spain, in accordance with the European Commission (2010/63/EU), FELASA and ARRIVE guidelines.

### Primary rat hippocampal cultures

Primary hippocampal cell cultures from Wistar rat E18 embryos were prepared as described before^60^. In the low-astrocyte cultures, cells were incubated with cytosine AraC (Sigma-Aldrich, Sintra, Portugal) at a concentration of 2 µM at DIV2–5. For cell viability assays, immunocytochemistry, Ca^2+^imaging, and electrophysiological recordings, cells were plated in glass coverslips at a density of 12,500 cells/cm^2^. Cells were used both at DIV7 and DIV14, except for synaptic density (morphological and electrophysiological analysis) and SBDP immunoreactivity evaluation performed only at DIV14, and in the cell viability assay in low-astrocyte cultures performed only at DIV7.

### In vitro cell viability assays

Rat hippocampal neurons were exposed to glutamate (1–100 µM) or NMDA (100 μM) during 30 min or 12 h. The different drugs tested were added 15 min before glutamate/NMDA exposure and were present until the end of the experiment. Cell viability was then assessed at different periods after the exposure of glutamate up to 24 h using a double staining with the fluorescent probes propidium iodide (PI) and SYTO-13 (Molecular Probes, Alfagene, Carcavelos, Portugal). Briefly, cells were loaded for 3 min in Krebs buffer (in mM: NaCl 132, KCl 4, MgCl_2_ 1.4, CaCl_2_ 1, glucose 6, HEPES-Na 10, pH 7.4) containing 4 μM SYTO-13 and 4 μg/mL PI. Cells were then visualized and images were acquired using an inverted fluorescence Zeiss Imager.Z2 microscope and the Axiovision SE64 Rel.4.8 software (Zeiss, Grupo Taper, Sintra, Portugal). Viable neurons present nuclei homogenously labeled with Syto-13 (green fluorescent nuclei). In contrast, putative apoptotic neurons show condensed and fragmented nuclei labeled with Syto-13 (primary apoptosis) or with Syto-13 plus PI (secondary apoptosis), and necrotic neurons present intact nuclei labeled with PI (dark, homogeneous, large red fluorescent nuclei). Each experiment in different cell cultures were performed in triplicate and cell counting was carried out in six fields per coverslip analyzing a minimum number of 300 cells per coverslip. Neuronal death was expressed as percentage of non-viable cells compared to the total number of cells.

Cell viability was further assessed by Alamar Blue and 3-[4,5-dimethylthiazole-2-yl]-2,5-diphenyltetrazolium bromide (MTT) assays. Cultured rat hippocampal neurons were incubated with Alamar blue dye at 10% (v/v) (Invitrogen, Alfagene) or MTT (0.5 mg/mL). In the Alamar Blue assay, after 4 h incubation aliquots of the media were collected from each well and absorbance of either reduced (570 nm) or oxidized form (600 nm) was measured. The relative change in reduction in the Alamar Blue assay were considered equivalent to the relative change in cell viability, as it is proportional to the number of viable cells containing competent mitochondria. In the MTT assay, after 2 h incubation, the insoluble purple product formazan resulting from the reduction of MTT by NAD(P)H-dependent oxidoreductases present in cells with viable mitochondria was solubilized in dimethyl sulfoxide for 1 h at room temperature, under agitation and protected from light. The percentage of MTT reduced was measured by the difference between the absorbances at 570 and 600 nm read in a spectrophotometer (Spectramax Plus 384, Molecular Devices, UK). The percentage of reduction of MTT was calculated comparing the difference of the absorbance at 570 and at 600 nm of each sample. Control wells containing medium only (no cells) were used to subtract the background absorbance. Results are presented as percentage of control (wells incubated with vehicle).

### Extracellular ATP quantification

After the exposure of cultured hippocampal neurons to glutamate (100 µM, 30 min), 50 μL of culture medium were collected at different time points (3–24 h) from each well and mixed with 50 μL of the luciferin-luciferase bioluminescent ATP assay mix from Sigma (FLAAM). The light emitted was quantified using a luminometer (PerkinElmer Victor3, Villepinte, France) and ATP levels were quantified using a calibration curve of ATP standards. The quantification in each condition and time-point was performed in triplicates.

### Single-cell Ca^2+^ imaging

Rat hippocampal neurons were loaded with Fluo-4 AM (2 μM, 30 min at 37 °C; Molecular Probes, Alfagene) in the following medium (in mM): NaCl 160 mM, KCl 2.5, CaCl_2_ 1.8, glucose 10, HEPES 10, MgCl_2_ 2 (pH 7.2). The cells were then washed twice to remove remaining Fluo-4 AM and mounted in a small perfusion chamber (Warner Instruments, USA) in the stage of a Zeiss Axiovert 200 inverted fluorescence microscope (Zeiss) equipped with a cooled CCD camera (Roper Scientific, Tucson, AZ, USA) that allowed the acquisition of 12-bit images at a rate of 1 Hz using MetaFluor 5.0 software (Zeiss). Drugs were applied to the cells in the same Krebs medium, except for NMDA-induced Ca^2+^ transients in which MgCl_2_ was omitted. Time-course data represents the average of fluorescence intensity obtained in the regions of interest.

### Electrophysiological recordings

NMDA-induced currents were recorded in rat hippocampal neurons at −60 mV by whole-cell patch clamping using an AxonPatch 200B amplifier (Molecular Devices) or List EPC-7 amplifier. The borosilicate glass micropipettes used had a resistance of 4–6 MΩ and were filled with the following internal solution (in mM): CsMeSO_4_ 130, CsCl 10, CaCl_2_ 0.5, EGTA 5, HEPES 10, and NaCl 10 (pH 7.3 adjusted with CsOH). Cells were perfused with extracellular solution containing 160 mM NaCl, 2.5 mM KCl, 1.8 mM CaCl_2_, 10 mM HEPES, 15 mM glucose, and 10 µM glycine (pH 7.4 adjusted with NaOH). All drugs were daily prepared in extracellular solution and rapidly perfused with a six channel perfusion valve control system VC-77SP/perfusion fast-step SF-77B (Warner Instruments, USA). The recordings of mEPSCs were performed at −70 mV using an internal solution containing the following (in mM): CsMeSO_4_ 125, NaCl 8, HEPES 10, EGTA 5, Qx-314 1, Mg-ATP 4, and Na-GTP 0.5 (pH 7.2). The bathing solution contained the following (in mM): NaCl 140, KCl 2.5, CaCl_2_ 1.8, MgCl_2_ 1, HEPES 10 and glucose 15 (pH 7.4) plus 1 μM tetrodotoxin, and 100 μM picrotoxin (Tocris, UK). All experiments were performed at RT (22–25 °C). The currents were filtered at 1–10 kHz (2-pole Butterworth filter, −12 dB/octave, or low-pass Bessel filter) and digitized at a sampling rate of 20 kHz to a personal computer to be analyzed with pClamp software (AXON Instruments, USA).

### KA-induced neurodegeneration in vivo

Male Wistar rats (weighting 250–300 g) were subjected to intraperitoneal administration of KA (10 mg/kg) or vehicle (0.9% NaCl). Fifteen minutes before, the animals were administered with a single i.c.v. injection of MRS2500 (1 nmol; Tocris) or artificial cerebrospinal fluid (in mM: NaCl 124, KCl 3, NaH_2_PO_4_ 1.25, NaHCO_3_ 26, MgCl_2_ 1, CaCl_2_ 2 and glucose 10) through a guide cannula previously implanted into the lateral ventricle at the following coordinates relative to Bregma: −0.8 mm antero-posterior; −1.5 mm lateral, and −3.5 mm dorso-ventral. Administration was done at the rate of 0.5 μL/min. At the end of the injection the needle remained in place for 3 min before being slowly removed from the cannula, to avoid reflux. This dose of MRS2500 has been previously validated in an in vivo model of brain ischemia^15^.

Seizure behavior was monitored for a 2-h period. Seizures induced by KA (10 mg/kg, Sigma) were classified into five stages according to the Racine’s scale^27^: stage I—facial automatisms; stage II—head nodding; stage III—forelimb clonus and lordotic posture; stage IV—forelimb clonus as the animal rears; stage V—forelimb clonus and rearing with falling over or loss of the righting reflex. Stages III–V are considered motor or convulsive seizures and should occur until 2 h after KA administration to guarantee hippocampal neurodegeneration^25^. Hence, only the animals reaching at least stage III of the Racine’s scale were subjected to histological or biochemical analysis 24 h later. For Western blot analysis, hippocampi were removed and homogenized in a 0.32 M sucrose solution containing 1 mM EDTA, 10 mM HEPES, and 1 mg/mL bovine serum albumin (BSA) (pH 7.4) at 4 °C. The homogenates were centrifuged at 300 × *g* for 10 min at 4 °C. The pellet was discarded and the supernatant was further centrifuged at 100,000 × *g* for 30 min at 4 °C. The supernatant was discarded and the pellet resuspended in 5% sodium dodecyl sulfate (SDS) solution with protease inhibitors (Roche Diagnostics, Amadora, Portugal).

### QA-induced neurodegeneration

Male Wistar rats (weighting 250–300 g) were placed in a stereotaxic frame (Stoelting) connected to an anesthesia system (EZ-7000 Classic System from E-Z Anesthesia, Plexx B.V.) containing isoflurane (ISOFLO^®^, Esteve Veterinaria, Spain), which maintained the animals anesthetized only during the stereotaxic procedure. Each animal received a single unilateral intrahippocampal injection (coordinates relative to Bregma: antero-posterior −3.5 mm, lateral −2.5 mm, and dorso-ventral −4.0 mm) of 1 µL at the rate of 0.2 µL/min controlled by an automatic injector system (syringe pump, model 11 plus, Harvard Apparatus), either of aCSF or of 120 nmol of QA. Within each group, some of the animals were co-administered 1 nmol of MRS2500. The osmolality of all of the injected solutions was adjusted with NaCl. The animals were monitored for convulsions for 2 h after the intrahippocampal injections and the histological evaluation was performed 24 h later.

### FJC staining

For histological evaluation of the hippocampal neurodegeneration by FJC staining, brains were fixed through transcardial perfusion and cut into 20-μm coronal sections comprising the whole hippocampus, using a cryostat (CM3050 S from Leica Microsystems, Carnaxide, Portugal). Brain slices were organized into series of 12 sections separated successively by 240 µm, each series being representative of all parts of the hippocampus. Brain sections were defrost, dried, and immersed for 5 min in 0.01% NaOH prepared in an 80% ethanol solution. After rising for 2 min in 70% ethanol and for 2 min in distilled water, slices were transferred to a 0.06% potassium permanganate solution during 10 min, under constant agitation and protected from light. Sections were again rinsed in distilled water for 2 min and immersed in 0.0001% FJC (Histo-Chem Inc., USA) in 0.1% of acetic acid for 10 min, protected from light and under agitation. After three washing steps of 1 min with distilled water, slices were dried on a slide warmer, dehydrated in an ethanol gradient (50%, 70%, and 100%), and cleared in xylene. Slices were then mounted in DPX non-aqueous mounting medium (Merck, Algés, Portugal). Images were acquired in a Zeiss Imager.Z2 microscope using the Axiovision SE64 Rel. 4.8 software. FJC-positive cells of complete series containing the hippocampus of each rat (12 sections per series) were quantified using the cell counter of the ImageJ 1.48v software.

### Immunocytochemistry analysis

Rat hippocampal neurons were fixed with 4% paraformaldehyde in 0.9% NaCl and 4% sucrose solution for 30 min and permeabilized with PBS 0.2% Triton X-100 for 10 min. The cells were then blocked with PBS 3% BSA for 30 min to prevent non-specific binding and then incubated with the primary antibodies in blocking solution for 1 h at room temperature. Hippocampal neurons were immunolabeled with a rabbit polyclonal anti-P2Y1R (1:200, Abcam, UK, Cat#ab104616 RRID:AB_10863775), mouse monoclonal anti-synaptophysin (1:200, Sigma, Cat#S5768, RRID:AB_477523), mouse monoclonal anti-SMI31 (1:1000, Covance; LusoPalex, Sintra, Portugal, Cat#SMI-31R-100, RRID:AB_10122491), chicken polyclonal anti-MAP2 (1:2500, Abcam, Cat#ab92434, RRID:AB_2138147) and/or rabbit polyclonal antibody directed against calpain-mediated SBDPs (1:300; Bahr et al.^36^). Next, the cells were incubated with AlexaFluor-488-conjugated donkey anti-mouse (1:400), AlexaFluor-555-conjugated goat anti-rabbit (1:400), and AlexaFluor-633-conjugated goat anti-chicken (1:400) in blocking solution for 1 h at room temperature. Coverslips were then mounted on glass slides using DAPI (4',6-diamidino-2-phenylindole)-supplemented mounting medium (Vectashield, Vector Laboratories, Baptista Marques, Lisboa, Portugal), cells were visualized using a laser scanning Zeiss LSM 510 Meta confocal microscope and images processed with ImageJ 1.48v software. Quantitative morphological analysis of the density of synaptophysin puncta per dendritic length was performed using Neurolucida software (MBF Bioscience, Netherlands) analyzing a minimum of 2000 µm of MAP2^+^ structures from two coverslips per condition per culture. The fluorescence intensity measurements of SMI31, SBDPs, and MAP2 were performed using ImageJ software. The fluorescence intensities from the 8-bit images (256-color scale) were directly converted into a 0–1 scale, being the absence of fluorescence set as 0 and the maximum intensity set as 1.

### Western blot analysis

Protein extracts from cultured hippocampal neurons or hippocampal homogenates were diluted in 6× sodium dodecyl sulfate-polyacrylamide gel electrophoresis (SDS-PAGE) sample buffer ((0.5 M Tris, 0.4% SDS, pH 6.8), 30% glycerol, 10% SDS, 0.6 M dithiothreitol, and 0.012% of bromophenol blue), boiled at 95 °C for 5 min and then separated by 10% SDS-PAGE electrophoresis. After the electrotransfer of the proteins, CRMP2 protein was detected using a rabbit antibody directed against CRMP2 (1:500; Abcam, Cat#ab36201, RRID:AB_731750) and the different synaptic markers were detected using mouse monoclonal antibodies directed against PSD-95 (1:20,000; Millipore, Merck, Cat#P246, RRID:AB_260911), synaptophysin (1:2000–1:20,000; Sigma, Cat#S5768, RRID:AB_477523), and syntaxin 1 A (1:2000; Sigma, Cat#S0664, RRID:AB_477483), guinea-pig polyclonal anti-vGluT1 (1:10,000; Synaptic Systems, Goettingen, Germany, Cat#135304 RRID:AB_887878), anti-vGAT (1:1000; Synaptic Systems; Cat#131004, RRID:AB_887873), and rabbit polyclonal anti-gephyrin (1:10,000; Abcam, Cat#ab25784 RRID:AB_1209349), all diluted in TBS-T (Tris-buffered saline, 0.1% Tween 20) containing 5% dried milk. Spectrin was detected using a mouse antibody directed against Spectrin α-chain (1:500; Millipore, Cat#MAB1622, RRID:AB_94295) diluted in TBS-T containing 5% BSA. A rabbit antibody directed against GAPDH (glyceraldehyde 3-phosphate dehydrogenase) (1:1000; Abcam, Cat#ab9485 RRID:AB_307275) and mouse monoclonal antibodies against α-tubulin (1:20 000; Sigma, Cat#T6074 RRID:AB_477582) or β-actin (1:20,000; Sigma, Cat#A5316 RRID:AB_476743) were used as loading controls. The membranes were incubated with horseradish peroxidase (HRP)-conjugated goat anti-rabbit or goat anti-mouse or goat anti-guinea-pig secondary antibodies (1:10,000; Pierce) and subsequently with SuperSignal West Pico Chemiluminescent Substrate (Pierce, Alfagene) or with Luminata Forte Western HRP Substrate (Millipore). The immunoreactivity was visualized using a VersaDoc3000 apparatus and analyzed using the ImageLab software (BioRad, Amadora, Portugal).

### [^3^H]Aspartic acid-binding assay

Percoll-purified synaptosomes prepared as previously described^18^ were resuspended in 15 mL hyposmolar binding assay medium (Tris/HCl, 50 mM; MgCl_2_, 3 mM; CaCl_2_, 1 µM; Na_2_EDTA, 2 mM; protease inhibitor cocktail (Sigma), 1 µL/mL, pH 7.4), and centrifuged at 20,000 *g* for 40 min to allow the separation of the synaptosomal membranes. Next, these synaptosomal membranes were diluted in the assay medium to reach ~1 mg/mL protein concentration, and 100 µL aliquots of this suspension were mixed with 100 µL assay medium with or without the selective antagonist of NMDARs D-AP-5 (30 μM, Tocris) to determine non-NMDAR binding, and with or without one of the following two selective antagonists of P2Y1R, MRS2179 (10 µM), or MRS2500 (10 µM) (Tocris). Finally, the 200 µL protein–receptor ligand mix was combined with a third aliquot of 100 µL containing [^3^H]aspartic acid (specific activity: 11.3 mCi/mM; PerkinElmer). The tested final concentrations (3–300 nM) of [^3^H]aspartic acid were chosen based on a previous NMDAR binding study in rat brain^61^. Each drug combination paired with each radioligand concentration was carried out in duplicate in 6–7 rats. After leaving the 300 µL mixtures in the bottom of 15 mL volume glass test tubes for 30 min at room temperature, they were rapidly rinsed in 10 mL ice-cold assay medium, and instantly vacuum-filtered onto Whatman GF/B borosilicate filters (Sigma). Filters were then collected and the radioactivity retention was counted with a Tricarb β-counter (PerkinElmer). *B*_max_ and *K*_D_ were calculated from the specific binding data with the help of GraphPad Prism.

### Statistical analysis

All data are presented as the mean ± SEM of *n* experiments. Group differences were analyzed using a two-tailed unpaired *t* test (comparison of two independent groups) or one-way analysis of variance (ANOVA) (comparison of several independent groups). Where ANOVA revealed significant general group effects, Dunnet’s post hoc test was used to compare different groups with the control group and Sidak’s post hoc test were used for multiple comparisons. Modifications in the frequency of mEPSCs were analyzed by Kruskal–Wallis test with Dunn’s multiple comparison post hoc test. The effect of the MRS2500 in the KA-induced cleavage of spectrin and CRMP2 was appraised by two-way ANOVA. The comparison of the impact of MRS2500 in the cell death induced by the exposure for 30 min and for 12 h to NMDA (100 µM) was evaluated by two-way ANOVA with Sidak’s multicomparison test. The extracellular levels of ATP in culture, the protein densities determined in Western blot analysis and the MTT reduction were expressed either as the fold change or relative percentage vs. control conditions. These relative differences were analyzed by one-sample *t* test by comparison with a hypothetical value of 1 or 100 (control condition). The average fluorescence intensities of SMI31 in segments without vs. with SBDP immunoreactivity were analyzed with Mann–Whitney test. In all analyses performed, a family-wise 95% confidence level (*p* < 0.05) was applied. All data processing and analyses were performed using the Prism 6.0 software (GraphPad).

## References

[1] Ikonomidou C, Turski L (1995). Excitotoxicity and neurodegenerative diseases. Curr. Opin. Neurol..

[2] Lipton SA, Rosenberg PA (1994). Excitatory amino acids as a final common pathway for neurologic disorders. N. Engl. J. Med..

[3] Lewerenz J, Maher P (2015). Chronic glutamate toxicity in neurodegenerative diseases—what is the evidence?. Front. Neurosci..

[4] Choi DW (1994). Calcium and excitotoxic neuronal injury. Ann. NY Acad. Sci..

[5] Rothman SM, Olney JW (1995). Excitotoxicity and the NMDA receptor—still lethal after eight years. Trends Neurosci..

[6] Vanderklish PW, Bahr BA (2000). The pathogenic activation of calpain: a marker and mediator of cellular toxicity and disease states. Int. J. Exp. Pathol..

[7] Dawson VL, Dawson TM (2004). Deadly conversations: nuclear-mitochondrial cross-talk. J. Bioenerg. Biomembr..

[8] Uttara B, Singh AV, Zamboni P, Mahajan RT (2009). Oxidative stress and neurodegenerative diseases: a review of upstream and downstream antioxidant therapeutic options. Curr. Neuropharmacol..

[9] Zhou Q, Sheng M (2013). NMDA receptors in nervous system diseases. Neuropharmacology.

[10] Yildiz-Unal A, Korulu S, Karabay A (2015). Neuroprotective strategies against calpain-mediated neurodegeneration. Neuropsychiatr. Dis. Treat..

[11] Rodrigues RJ, Tomé AR, Cunha RA (2015). ATP as a multi-target danger signal in the brain. Front. Neurosci..

[12] Burnstock G, Krügel U, Abbracchio MP, Illes P (2011). Purinergic signalling: from normal behaviour to pathological brain function. Prog. Neurobiol..

[13] Sperlagh B, Illes P (2014). P2X7 receptor: an emerging target in central nervous system diseases. Trends Pharmacol. Sci..

[14] Kuboyama K (2011). Astrocytic P2Y1 receptor is involved in the regulation of cytokine/chemokine transcription and cerebral damage in a rat model of cerebral ischemia. J. Cereb. Blood Flow Metab..

[15] Carmo MR (2014). ATP P2Y1 receptors control cognitive deficits and neurotoxicity but not glial modifications induced by brain ischemia in mice. Eur. J. Neurosci..

[16] Choo AM (2013). Antagonism of purinergic signalling improves recovery from traumatic brain injury. Brain.

[17] Chin Y (2013). Involvement of glial P2Y1 receptors in cognitive deficit after focal cerebral stroke in a rodent model. J. Neuroinflamm..

[18] Rodrigues RJ, Almeida T, Richardson PJ, Oliveira CR, Cunha RA (2005). Dual presynaptic control by ATP of glutamate release via facilitatory P2X1, P2X2/3, and P2X3 and inhibitory P2Y1, P2Y2, and/or P2Y4 receptors in the rat hippocampus. J. Neurosci..

[19] Mendonza-Fernandez V, Andrew RD, Barajas-López C (2000). ATP inhibits glutamate synaptic release by acting at P2Y receptors in pyramidal neurons of hippocampal slices. J. Pharmacol. Exp. Ther..

[20] Luthardt J (2003). P2Y(1) receptor activation inhibits NMDA receptor-channels in layer V pyramidal neurons of the rat prefrontal and parietal cortex. Neurochem. Int..

[21] Gerevich Z (2004). Inhibition of N-type voltage-activated calcium channels in rat dorsal root ganglion neurons by P2Y receptors is a possible mechanism of ADP-induced analgesia. J. Neurosci..

[22] Filippov AK, Choi RCY, Simon J, Barnard EA, Brown DA (2006). Activation of P2Y1 nucleotide receptors induces inhibition of the M-type K+ current in rat hippocampal pyramidal neurons. J. Neurosci..

[23] Guzman SJ (2010). P2Y1 receptors inhibit long-term depression in the prefrontal cortex. Neuropharmacology.

[24] Traini C (2011). P2 receptor antagonists prevent synaptic failure and extracellular signal-regulated kinase 1/2 activation induced by oxygen and glucose deprivation in rat CA1 hippocampus in vitro. Eur. J. Neurosci..

[25] Ben-Ari Y (1985). Limbic seizure and brain damage produced by kainic acid: mechanisms and relevance to human temporal lobe epilepsy. Neuroscience.

[26] Wang Q, Yu S, Simonyi A, Sun GY, Sun AY (2005). Kainic acid-mediated excitotoxicity as a model for neurodegeneration. Mol. Neurobiol..

[27] Racine RJ (1972). Modification of seizure activity by electrical stimulation. II. Motor seizure. Electroencephalogr. Clin. Neurophysiol..

[28] Bi X, Chang V, Siman R, Tocco G, Baudry M (1996). Regional distribution and time-course of calpain activation following kainate-induced seizure activity in adult rat brain. Brain. Res..

[29] Araujo IM (2008). Calpain activation is involved in early caspase-independent neurodegeneration in the hippocampus following status epilepticus. J. Neurochem..

[30] Bretin S (2006). Calpain product of WT-CRMP2 reduces the amount of surface NR2B NMDA receptor subunit. J. Neurochem..

[31] Berg M, Bruh T, Johansen FF, Diemer NH (1993). Kainic acid-induced seizures and brain damage in the rat: different effects of NMDA- and AMPA receptor antagonists. Pharmacol. Toxicol..

[32] Bowser DN, Khakh BS (2004). ATP excites interneurons and astrocytes to increase synaptic inhibition in neuronal networks. J. Neurosci..

[33] del Puerto A (2012). Adenylate cyclase 5 coordinates the action of ADP, P2Y1, P2Y13 and ATP-gated P2X7 receptors on axonal elongation. J. Cell Sci..

[34] Higuchi M (2005). Distinct mechanistic roles of calpain and caspase activation in neurodegeneration as revealed in mice overexpressing their specific inhibitors. J. Biol. Chem..

[35] del Cerro S (1994). Stimulation of NMDA receptors activates calpain in cultured hippocampal slices. Neurosci. Lett..

[36] Bahr BA, Tiriveedhi S, Park GY, Lynch G (1995). Induction of calpain-mediated spectrin fragments by pathogenic treatments in long-term hippocampal slices. J. Pharmacol. Exp. Ther..

[37] Melo CV (2013). Spatiotemporal resolution of BDNF neuroprotection against glutamate excitotoxicity in cultured hippocampal neurons. Neuroscience.

[38] Ma M (2013). Role of calpains in the injury-induced dysfunction and degeneration of the mammalian axon. Neurobiol. Dis..

[39] Lepeta K (2016). Synaptopathies: synaptic dysfunction in neurological disorders—a review from students to students. J. Neurochem..

[40] Domercq M (2006). P2Y1 receptor-evoked glutamate exocytosis from astrocytes: control by tumor necrosis factor-alpha and prostaglandins. J. Biol. Chem..

[41] Jourdain P (2007). Glutamate exocytosis from astrocytes controls synaptic strength. Nat. Neurosci..

[42] Jarrard LE (2002). Use of excitotoxins to lesion the hippocampus: update. Hippocampus.

[43] Nadler JV, Perry BW, Cotman CW (1978). Intraventricular kainic acid preferentially destroys hippocampal pyramidal cells. Nature.

[44] Lerma J, Marques JM (2013). Kainate receptors in health and disease. Neuron.

[45] Sattler R, Charlton MP, Hafner M, Tymianski M (1998). Distinct influx pathways, not calcium load, determine neuronal vulnerability to calcium neurotoxicity. J. Neurochem..

[46] Hardingham GE, Fukunaga Y, Bading H (2002). Extrasynaptic NMDARs oppose synaptic NMDARs by triggering CREB shut-off and cell death pathways. Nat. Neurosci..

[47] Zhou X, Ding Q, Chen Z, Yun H, Wang H (2013). Involvement of the GluN2A and GluN2B subunits in synaptic and extrasynaptic *N*-methyl-d-aspartate receptor function and neuronal excitotoxicity. J. Biol. Chem..

[48] Stanika RI (2009). Coupling diverse routes of calcium entry to mitochondrial dysfunction and glutamate excitotoxicity. Proc. Natl. Acad. Sci. USA.

[49] Suárez LM (2005). Presynaptic NMDA autoreceptors facilitate axon excitability: a new molecular target for the anticonvulsant gabapentin. Eur. J. Neurosci..

[50] Hosie KA, King AE, Blizzard CA, Vickers JC, Dickson TC (2012). Chronic excitotoxin-induced axon degeneration in a compartmented neuronal culture model. ASN Neuro..

[51] Raff MC, Whitmore AV, Finn JT (2002). Axonal self-destruction and neurodegeneration. Science.

[52] Coleman MP, Perry VH (2002). Axon pathology in neurological disease: a neglected therapeutic target. Trends Neurosci..

[53] Neukomm LJ, Freeman MR (2014). Diverse cellular and molecular modes of axon degeneration. Trends Cell Biol..

[54] Saxena S, Caroni P (2007). Mechanisms of axon degeneration: from development to disease. Prog. Neurobiol..

[55] Gould TW (2006). Complete dissociation of motor neuron death from motor dysfunction by Bax deletion in a mouse model of ALS. J. Neurosci..

[56] Ferri A, Sanes JR, Coleman MP, Cunningham JM, Kato AC (2003). Inhibiting axon degeneration and synapse loss attenuates apoptosis and disease progression in a mouse model of motoneuron disease. Curr. Biol..

[57] Finn JT (2000). Evidence that Wallerian degeneration and localized axon degeneration induced by local neurotrophin deprivation do not involve caspases. J. Neurosci..

[58] Berliocchi L (2005). Botulinum neurotoxin C initiates two different programs for neurite degeneration and neuronal apoptosis. J. Cell Biol..

[59] Villmann C, Becker CM (2007). On the hypes and falls in neuroprotection: targeting the NMDA receptor. Neuroscientist.

[60] Rodrigues RJ (2016). Presynaptic P2X1-3 and α3-containing nicotinic receptors assemble into functionally interacting ion channels in the rat hippocampus. Neuropharmacology.

[61] Monaghan DT, Cotman CW (1985). Distribution of *N*-methyl-d-aspartate-sensitive l-[^3^H]glutamate-binding sites in rat brain. J. Neurosci..

